# Social media addiction and social anxiety among college students: the serial mediating roles of alienation, self-focused attention, and fear of negative evaluation

**DOI:** 10.3389/fpsyg.2026.1797966

**Published:** 2026-04-07

**Authors:** Xiaoyu Xu, Hao Fang

**Affiliations:** Zhejiang Agricultural Business College, Shaoxing, China

**Keywords:** alienation, fear of negative evaluation, self-focused attention, social anxiety, social media addiction

## Abstract

**Background:**

Previous studies have established a correlation between social media addiction and social anxiety among college students, but the mechanisms underlying this relationship remain incompletely understood. This study aimed to investigate the serial mediating effects of alienation, self-focused attention, and fear of negative evaluation in the relationship between social media addiction and social anxiety.

**Methods:**

A total of 541 college students (mean age = 19.40, SD = 1.30) completed the Bergen Social Media Addiction Scale, the Interaction Anxiousness Scale, the General Alienation Scale, the Brief Fear of Negative Evaluation Scale, and the Self-Focused Attention Questionnaire.

**Results:**

The results indicate that alienation, self-focused attention, and fear of negative evaluation serve as serial mediators in the relationship between social media addiction and social anxiety. In other words, these variables sequentially and collectively mediate the effect of social media addiction on social anxiety. Furthermore, the study reveals that the relationship between social media addiction and social anxiety is fully mediated by alienation, self-focused attention, and fear of negative evaluation.

**Conclusion:**

The findings suggest that social media addiction is associated with feelings of alienation, which in turn are linked to heightened self-focused attention. Sustained self-focused attention is further associated with increased fear of negative evaluation, which is related to social anxiety. This model illustrates a potential pathway through which these variables are interconnected.

## 1 Introduction

### 1.1 Social media addiction and social anxiety

In today's highly interconnected digital society, social media addiction has become a prevalent behavioral phenomenon. It is characterized by individuals' excessive cognitive, emotional, and behavioral engagement with social media, including difficulty controlling the urge to use it, a significant increase in usage time, and withdrawal reactions such as irritability when unable to access it (Andreassen, 2015; Andreassen et al., 2016; Bányai et al., 2017; Cheng et al., 2021; Kuss and Griffiths, 2011; Smith, 2023; Sun and Zhang, 2021). Concurrently, the prevalence of social anxiety among young people has demonstrated a consistent upward trend. The core features of social anxiety involve intense fear or anxiety in social or performance situations, accompanied by persistent concern that one's actions or words may elicit negative evaluation from others, often resulting in observable social avoidance or psychological distress (American Psychiatric Association, 2022; Hofmann, 2007). Although extensive cross-sectional research has established a positive correlation between excessive social media use and negative emotional symptoms such as depression, anxiety, and loneliness (Andreassen et al., 2017; Boer et al., 2021; Caplan, 2005, 2007; Lin et al., 2016; Primack et al., 2017; Zhang et al., 2025; Zhao et al., 2022), the causal relationship and underlying mechanisms between social media addiction and social anxiety remain unclear. This gap in knowledge directly impedes the development of effective prevention and intervention strategies. Notably, increasing concern among both the public and academic communities regarding “social media addiction” and its psychosocial impact underscores the urgency and theoretical significance of conducting more precise and in-depth investigations into its underlying psychological mechanisms.

Hypothesis 1: social media addiction positively predicts social anxiety (H1).

### 1.2 Alienation

The digital media environment, particularly its virtual and asynchronous nature of interactions, can profoundly influence users' socio-emotional experiences. Alienation, in the context of digital media, refers to the perceived disconnection and emotional distance from one's authentic self, others, and the social environment in the physical world, which occurs while immersed in highly connected virtual networks (Twenge et al., 2021). Theoretically, excessive reliance on curated online interactions and self-presentation may reduce an individual's ability to form deep, authentic interpersonal relationships, resulting in superficial offline socialization and a lack of meaningful emotional support. Research indicates that excessive social media use is significantly correlated with perceived social isolation (Primack et al., 2017), and such isolation and emotional detachment have been identified as strong predictors of depressive and anxious moods (Hunt et al., 2018; Schinka et al., 2012). Consequently, social media addiction may heighten an individual's susceptibility to social anxiety by intensifying feelings of alienation.

Hypothesis 2: alienation acts as a mediator between social media addiction and social anxiety (H2).

### 1.3 Self-focused attention

Alienation, which may be induced or reinforced by the media environment, could further lead to specific changes in cognitive patterns. Among these, self-focused attention refers to the cognitive tendency to persistently and repeatedly direct attentional resources toward one's internal states and externally observable aspects of the self (Clark and Wells, 1995). In social situations, excessive self-focus leads individuals to over-monitor and evaluate their own performance, thereby interfering with the normal attention paid to situational cues and others, while amplifying the perception of potential social threats (Ingram, 1990). Self-regulation theory posits that when individuals perceive a discrepancy between their actual self and their ideal standards, self-focused attention readily induces anxiety (Carver and Scheier, 1981). Furthermore, social media platforms emphasize self-presentation, image management, and social comparison (Fox and Vendemia, 2016; Nadkarni and Hofmann, 2012), potentially reinforcing this persistent self-scrutiny unconsciously.

Hypothesis 3: self-focused attention acts as a mediator between social media addiction and social anxiety (H3).

### 1.4 Fear of negative evaluation

The cognitive pattern of excessive self-focus naturally directs individuals toward heightened sensitivity to social evaluation. Fear of negative evaluation, as the most central cognitive feature of social anxiety, is characterized by anticipatory, pervasive, and intense apprehension regarding potential criticism, rejection, or negative appraisal by others (Weeks et al., 2005). This fear extends beyond normative social concerns and frequently results in pronounced avoidance of evaluative situations or substantial psychological distress. Cognitive-behavioral models posit that its etiology stems from maladaptive belief systems, such as the conviction that “I must be accepted by everyone” (Ahmed and Vaghefi, 2021; Clark and Wells, 1995). The contemporary media environment, through its capacity to quantify, visualize, and instantaneously disseminate social feedback, may substantially intensify individuals' sensitivity to and reliance upon others' evaluations, thereby reinforcing and exacerbating this fear. When an individual's self-worth becomes disproportionately contingent upon online feedback, the fear of negative evaluation may readily generalize from digital interactions to broader social functioning (Wang et al., 2018).

Hypothesis 4: fear of negative evaluation acts as a mediator between social media addiction and social anxiety (H4).

Building on the cognitive-behavioral model of social anxiety (Clark and Wells, 1995), it is worth further exploring whether the three key psychological variables—alienation, self-focused attention, and fear of negative evaluation—do not operate independently, but rather form a sequential chain in the process linking social media addiction to social anxiety. According to this theoretical framework, maladaptive cognitive patterns and dysfunctional beliefs about evaluation are central to the maintenance of social anxiety. The digital media environment may activate and reinforce these cognitive vulnerabilities through a specific developmental sequence. Specifically, addiction may first reduce an individual's real-world sense of belonging, triggering alienation. This emotional isolation can then cause individuals to turn inward, heightening their focus on internal states—that is, increasing self-focused attention. When attention is fixated on the self, self-related information becomes prioritized in cognition. This state directly catalyzes and intensifies concerns about how one is perceived by others, thereby amplifying the fear of negative evaluation. Ultimately, this persistent fear and avoidance of negative evaluation likely constitutes the core experience of social anxiety (American Psychiatric Association, 2022). This hypothesis outlines a potential psychological progression from behavioral dependence to emotional disorder, grounded in established cognitive-behavioral principles.

Hypothesis 5: alienation and self-focused attention act as serial mediators between social media addiction and social anxiety (H5).

Hypothesis 6: alienation and fear of negative evaluation act as serial mediators between social media addiction and social anxiety (H6).

Hypothesis 7: self-focused attention and fear of negative evaluation act as serial mediators between social media addiction and social anxiety (H7).

Hypothesis 8: alienation, self-focused attention, and fear of negative evaluation act as serial mediators between social media addiction and social anxiety (H8).

In summary, although prior research has separately established associations between social media addiction and alienation, self-focused attention, or fear of negative evaluation, most studies have treated these variables as independent mediators or have focused on bivariate correlations. Consequently, the existing literature lacks a unified theoretical framework to explain how these factors might operate sequentially and interdependently to transmit the effect of social media addiction onto social anxiety. While previous studies have identified simple mediation effects, they have not tested the theoretically driven sequential process proposed here. The present study addresses this gap by proposing and empirically testing an integrative serial mediation model grounded in the cognitive-behavioral framework of social anxiety (Clark and Wells, 1995). By examining the full sequential pathway, this study provides a more nuanced understanding of the mechanism linking behavioral addiction to emotional distress, thereby contributing to both theory development and the design of targeted interventions. Thus, this study aims to systematically test all the above hypotheses by constructing and examining this novel serial multiple mediation model, thereby offering a more comprehensive understanding of the underlying mechanisms.

## 2 Materials and methods

### 2.1 Participants

#### Recruitment feasibility and sample size determination

A power analysis was conducted using G^*^Power 3.1.9.7 (Faul et al., 2007) to determine the required sample size for the study. Based on previous research examining relationships between media use and psychological variables, a small effect size (correlation ρ H1 = 0.20) was specified (Cohen, 1988). With parameters set for a two-tailed test, an alpha error probability of 0.05, and a desired power (1–β) of 0.95, the analysis indicated that a minimum sample size of 319 participants was required.

#### Participant characteristics

The survey was conducted from December 2025 using an online questionnaire system (SoJump, a Chinese online survey tool). The survey link was distributed to participants via social media platforms. All participants were full-time undergraduate students in China and were required to be fluent in reading and understanding Chinese. Before starting the questionnaire, each participant was presented with an informed consent form explaining the study's purpose, the voluntary nature of participation, and the confidentiality of their responses. Only those who provided consent proceeded to the questionnaire. No incentives were offered. A total of 600 participants initially volunteered; 59 were excluded due to incomplete questionnaires or missing demographic information, yielding a final sample of 541 valid participants (validity rate of 90%). This final sample exceeded the minimum sample size determined by the power analysis.

Regarding demographic characteristics, 217 participants were male (40.1%) and 324 participants were female (59.9%). The grade distribution was as follows: 285 participants (52.7%) were freshmen, 164 participants (30.3%) were sophomores, 71 participants (13.1%) were juniors, and 21 participants (3.9%) were seniors. Their average age was 19.26 (SD = 1.30; age range = 18–22). No incentives were provided for participation in this study.

### 2.2 Measures

#### Bergen social media addiction scale

Social media addiction was measured using the Bergen Social Media Addiction Scale (Andreassen et al., 2016), employing the Chinese version revised by Chen et al. (2020). The scale consists of 6 items (e.g., “If I am unable to access social media, I feel distressed.”). Responses are scored on a 5-point Likert scale (1 = very rarely, 5 = very often). Higher total scores indicate a higher level of social media addiction. The Cronbach's alpha for this study was 0.93.

#### Interaction anxiousness scale

Social anxiety was measured using the Interaction Anxiousness Scale developed by Leary (1983), utilizing the Chinese version validated by Peng et al. (2004) for Chinese college students. The IAS consists of 15 items (e.g., “Even at informal gatherings, I often feel nervous”), each scored on a 5-point scale from 1 to 5 (1 = not at all characteristic, 5 = extremely characteristic). A higher score obtained from this scale was associated with a correspondingly higher level of social anxiety in participants. The higher the score on this scale, the higher the level of social anxiety. The Cronbach's alpha for this study was 0.77.

#### General alienation scale

Alienation was measured using the General Alienation Scale revised by Chen et al. (2015). The scale consists of 12 items (e.g., “I often feel that I cannot participate in what others are doing.”), rated on a 4-point Likert scale (1 = strongly disagree, 4=strongly agree). Higher total scores indicate a stronger sense of alienation. The Cronbach's alpha for this study was 0.87.

#### Brief fear of negative evaluation scale

Fear of negative evaluation was measured using the Brief Fear of Negative Evaluation Scale revised by Chen (2002). The scale includes 12 items (e.g., “I worry about what other people will think of me even when I know it doesn't make any difference.”), rated on a 5-point Likert scale (1 = not at all characteristic, 5 = extremely characteristic). Higher scores reflect a greater fear of negative evaluation. The Cronbach's alpha for this study was 0.69. While this value is slightly below the commonly cited threshold of 0.70 (Nunnally, 1978), it is considered acceptable for research purposes, particularly for scales with a relatively small number of items, and is consistent with reliabilities reported in some previous studies using this scale (Duke et al., 2006).

#### Self-focused attention questionnaire

Self-focused attention was measured using the Chinese Students Self-Focused Attention Questionnaire developed by Li and Lu (2018). The questionnaire comprises 21 items (e.g., “I feel a large gap between my actual self and my ideal self.”). Responses are scored on a 5-point Likert scale (1 = not at all characteristic, 5 = extremely characteristic). Higher total scores indicate a stronger tendency toward self-focused attention. The Cronbach's alpha for this study was 0.98.

### 2.3 Data analysis

Statistical analyses were conducted using SPSS 26.0 (IBMCorp., Armonk, N.Y., USA). Preliminary analyses included bivariate correlations to evaluate relationships among key variables. To examine potential common method bias, Harman's single-factor test was applied by including all variables in an exploratory factor analysis and reviewing the unrotated factor solution (Podsakoff et al., 2003).

To examine the hypothesized serial multiple mediation model, Model 6 of the PROCESS macro for SPSS (Version 4.3.1; Hayes, 2022) was used. The significance of indirect effects was tested via bootstrapping with 10,000 resamples, producing 95% bias-corrected confidence intervals (Bollen and Stine, 1990). Statistical significance was defined as *p* < 0.05.

## 3 Results

### 3.1 Common method bias test

For testing common method bias, Harman's single-factor test was mainly used (Podsakoff et al., 2003; Zhou and Long, 2004). Exploratory factor analysis was conducted on all variables included in the four questionnaires. From the results of the analysis, there were 13 factors with eigenvalues greater than one, while the variance explained by the first factor was much less than 40%, at 33.79%. The results suggested that common method bias does not exist. However, it is important to acknowledge that this test is a diagnostic tool and not a definitive remedy for CMB.

### 3.2 Descriptive statistics

Based on the correlation analysis (see Table 1), social media addiction showed significant positive correlations with social anxiety (*r* = 0.65, *p* < 0.01), alienation (*r* = 0.76, *p* < 0.01), fear of negative evaluation (*r* = 0.57, *p* < 0.01), and self-focused attention (*r* = 0.83, *p* < 0.01). Social anxiety was also positively correlated with fear of negative evaluation (*r* = 0.77, *p* < 0.01), self-focused attention (*r* = 0.84, *p* < 0.01), and alienation (*r* = 0.66, *p* < 0.01). Similarly, alienation exhibited significant positive correlations with fear of negative evaluation (*r* = 0.63, *p* < 0.01) and self-focused attention (*r* = 0.81, *p* < 0.01), and fear of negative evaluation was positively correlated with self-focused attention (*r* = 0.77, *p* < 0.01).

**Table 1:** Means, standard deviations, and correlations among the variables.

Variables	*M*	*SD*	1	2	3	4	5	6
1 Social media addiction	2.97	1.03	1					
2 Alienation	2.37	0.60	0.76^**^	1				
3 Self-focused attention	3.02	1.02	0.83^**^	0.81^**^	1			
4 Fear of negative evaluation	3.08	0.56	0.57^**^	0.63^**^	0.77^**^	1		
5 Social anxiety	3.12	0.62	0.65^**^	0.66^**^	0.84^**^	0.77^**^	1	

*N* = 541.

^*^p < 0.05,

^**^p < 0.01,

^***^p < 0.001.

We evaluated the potential for multicollinearity in the regression models. Collinearity diagnostics were conducted for each regression model in the serial mediation analysis. The variance inflation factor values for all predictors across all models ranged from 1.00 to 3.54, which are substantially below the commonly recommended threshold of 5 or 10 (Hair et al., 2010). Tolerance values all exceeded 0.28. These findings suggest that multicollinearity does not pose a significant concern in this study, and the regression coefficients were estimated with adequate stability.

**Figure 1:**
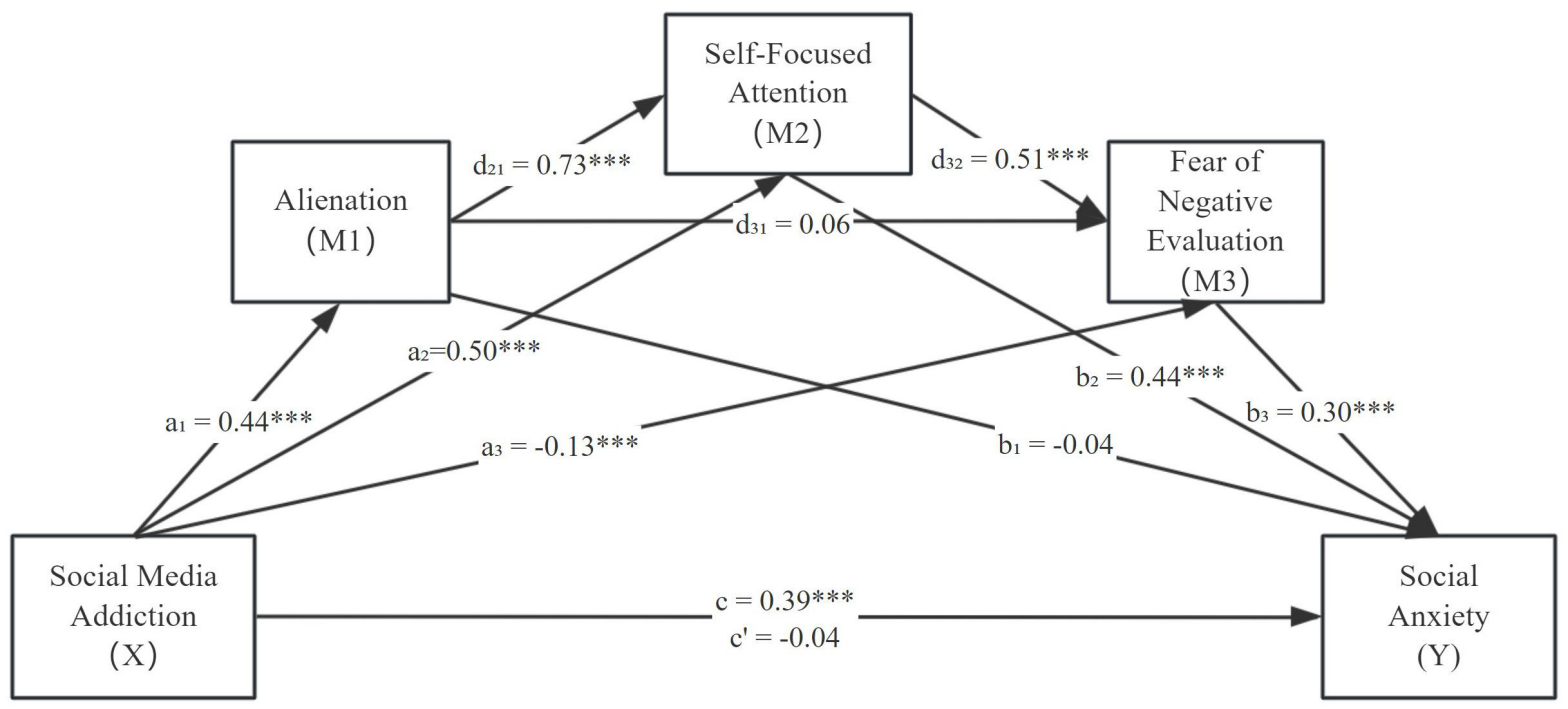
The serial multiple mediation analysis of the relationship between social media addiction and social anxiety, mediated through alienation, self-focused attention, and fear of negative evaluation. *c*: total effect of social media addiction; *c'*: direct effect of social media addiction after controlling for the mediators. Unstandardized regression coefficients are reported. *p < 0.05, **p < 0.01, ***p < 0.001.

Table 2 summarizes the results of the PROCESS analysis. Figure 1 show the results of the serial multiple mediation analyses.The total effect of social media addiction on social anxiety was significant (*c* = 0.39, SE = 0.02, *t* = 20.00, *p* < 0.001). However, after introducing alienation, self-focused attention, and fear of negative evaluation as mediators, the direct effect of social media addiction on social anxiety became non-significant (*c'* = −0.04, SE = 0.02, *t* = −1.75, *p* = 0.08), leading to the rejection of Hypothesis 1.

**Table 2:** Summary of the multiple regression analyses for the serial multiple mediation model.

Variables	Model 1					Model 2					Model 3					Model 4				
	*B*	95% CI	*SE B*	β	*t*	*B*	95% CI	*SE B*	β	*t*	*B*	95% CI	*SE B*	β	*t*	*B*	95% CI	*SE B*	β	*t*
Constant	1.06	[0.96, 1.17]	0.05		20.66^***^	−0.20	[−0.37, −0.03]	0.09		−2.31^*^	1.80	[1.67, 1.92]	0.06		28.98^***^	1.09	[0.91, 1.26]	0.09		12.19^***^
Social media addiction	0.44	[0.41, 0.47]	0.02	0.76	26.89^***^	0.50	[0.43, 0.56]	0.03	0.50	15.78^***^	−0.13	[−0.19, −0.08]	0.03	−0.25	−4.94^***^	−0.04	[−0.09, 0.01]	0.02	−0.07	−1.75
Alienation						0.73	[0.63, 0.84]	0.05	0.43	13.60^***^	0.06	[−0.03, 0.15]	0.04	0.06	1.29	−0.04	[−0.12, 0.04]	0.04	−0.04	−1.09
Self-focused attention											0.51	[0.45, 0.57]	0.03	0.93	16.64^***^	0.44	[0.38, 0.51]	0.03	0.72	13.00^***^
Fear of negative evaluation																0.30	[0.23, 0.38]	0.04	0.27	7.80^***^
Model summary	*R^2^* = 0.57, *F*_(1, 539)_ = 723.10, *p* < 0.001					*R^2^* = 0.77, *F*_(2, 538)_ = 888.73, *p* < 0.001					*R^2^* = 0.61, *F*_(3, 537)_ = 284.81, *p* < 0.001					*R^2^* = 0.74, *F*_(4, 536)_ = 391.03, *p* < 0.001				

Note: *N* = 541. Model 1: predictor variable: social media addiction; outcome variable: alienation. Model 2: predictor variables: social media addiction and alienation; outcome variable: self-focused attention. Model 3: predictor variables: social media addiction, alienation, and self-focused attention; outcome variable: fear of negative evaluation. Model 4: predictor variables: social media addiction, alienation, self-focused attention, and fear of negative evaluation; outcome variable: social anxiety

^*^p < 0.05,

^**^p < 0.01,

^***^p < 0.001.

Analysis of the indirect effects using the bootstrap method (with 10,000 resamples) supported the significance of the following paths (see Table 3): Path 2 (Social Media Addiction → Self-Focused Attention → Social Anxiety): *B* = 0.22, Boot SE = 0.04, 95% Boot CI [0.15, 0.30], supporting Hypothesis 3. Path 3 (Social Media Addiction → Fear of Negative Evaluation → Social Anxiety): *B* = −0.04, Boot SE = 0.01, 95% Boot CI [−0.07, −0.01], supporting Hypothesis 4. Path 4 (Social Media Addiction → Alienation → Self-Focused Attention → Social Anxiety): *B* = 0.14, Boot SE = 0.03, 95% Boot CI [0.09, 0.20], supporting Hypothesis 5. Path 6 (Social Media Addiction → Self-Focused Attention → Fear of Negative Evaluation → Social Anxiety): *B* = 0.08, Boot SE = 0.02, 95% Boot CI [0.05, 0.11], supporting Hypothesis 7. Path 7 (Social Media Addiction → Alienation → Self-Focused Attention → Fear of Negative Evaluation → Social Anxiety): *B* = 0.05, Boot SE = 0.01, 95% Boot CI [0.03, 0.07], supporting Hypothesis 8. However, Paths 1 and 5 were not significant, rejecting Hypothesis 2 and Hypothesis 6, respectively.

**Table 3:** Bootstrapping indirect effects and 95% CI for sequential mediation model.

Model pathway	Effect	Boot SE	95% CI	
			Lower	Upper
Path 1 social media addiction → alienation → social anxiety	−0.0193	0.0259	−0.0717	0.0295
Path 2 social media addiction → aelf-focused attention → social Anxiety	0.2194	0.0380	0.1520	0.2999
Path 3 social media addiction → fear of negative evaluation → social anxiety	−0.0405	0.0146	−0.0716	−0.0147
Path 4 social media addiction → alienation → self-focused attention → social anxiety	0.1431	0.0282	0.0939	0.2033
Path 5 social media addiction → alienation → fear of negative evaluation → social anxiety	0.0077	0.0089	−0.0089	0.0268
Path 6 social media addiction → self-focused attention → fear of negative evaluation → social anxiety	0.0768	0.0170	0.0451	0.1114
Path 7 social media addiction → alienation → self-focused attention → fear of negative evaluation → social anxiety	0.0501	0.0118	0.0285	0.0749
Total	0.4373	0.0403	0.3614	0.5202

*N* = 541. CI, Confidence interval; 10,000 bootstrap samples with 95% CI.

The results revealed a significant indirect effect of social media addiction on social anxiety through the serial mediation chain of alienation, self-focused attention, and fear of negative evaluation [total indirect effect = 0.44, Boot SE = 0.04, 95% Boot CI (0.36, 0.52)], supporting Hypothesis 8. Furthermore, the total effect of social media addiction on social anxiety in the presence of the mediators remained significant (*c* = 0.39, SE = 0.02, *p* < 0.001). Hence, alienation, self-focused attention, and fear of negative evaluation fully mediated the relationship between social media addiction and social anxiety.

## 4 Discussion

This study constructed and tested a serial multiple mediation model to examine the internal mechanisms of alienation, self-focused attention, and fear of negative evaluation in the relationship between social media addiction and social anxiety. The results indicated that while the total effect of social media addiction on social anxiety was significant, the direct effect became non-significant after introducing the three mediators: alienation, self-focused attention, and fear of negative evaluation. This finding demonstrates that the relationship between social media addiction and social anxiety is fully mediated by these factors rather than partially mediated. Consequently, Hypothesis 1, which posited a direct link, was not supported by the data. The influence of social media addiction on social anxiety should therefore be interpreted as entirely indirect, operating through the sequential psychological mechanisms proposed in this study rather than through a direct pathway.

This outcome contrasts with previous studies that have emphasized a direct predictive effect (Li and Fan, 2022; Tsomokos, 2025). The discrepancy may stem from the inclusion of core cognitive-affective variables more closely associated with the nature of social anxiety—specifically, self-focused attention and fear of negative evaluation. The cognitive model of social anxiety (Clark and Wells, 1995) and social cognitive theory (Bandura, 1986) propose that excessive self-focused attention and fear of negative evaluation are central maintaining factors. When these key mediating mechanisms were accounted for in the model, the direct effect of media-dependent behavior itself was no longer significant. This suggests that social media addiction does not directly induce anxiety but instead exerts its influence indirectly by fostering a psychological state prone to anxiety, characterized by interpersonal alienation, excessive self-monitoring, and fear of evaluation.

The core finding of this study validated the significance of the serial mediation chain from alienation to self-focused attention and subsequently to fear of negative evaluation. This pathway clearly demonstrates the potential progressive psychological impact of the digital media environment: excessive dependence on media may erode an individual's sense of connection to the real world, thereby triggering alienation (Twenge et al., 2021; Xu and He, 2025). This emotional isolation may, in turn, lead individuals to direct greater attention toward their internal states and self-presentation, consequently enhancing self-focused attention (Boehme et al., 2015; Clark and Wells, 1995). Sustained self-focus amplifies the monitoring and evaluation of self-performance in social situations, further intensifying the fear of potential negative evaluation from others (Weeks et al., 2005), which constitutes the core cognitive feature of social anxiety (American Psychiatric Association, 2022). Thus, this model provides a coherent, sequential theoretical framework for understanding the transition from maladaptive media use behavior to social anxiety.

It is noteworthy that the independent mediating role of alienation (H_2_) and the direct path from alienation to fear of negative evaluation (H6) were not supported. These results suggest that, within the framework of this study, alienation may not independently or directly influence anxiety by exacerbating fear of evaluation. Instead, its impact appears to be mediated through changes in individuals' cognitive attentional patterns. Only after such a shift occurs can the effect of alienation be transmitted to fear of negative evaluation, ultimately contributing to social anxiety. This finding highlights the sequential dependency among these psychological variables.

## 5 Limitations and future research

This study has several limitations. First, the current study employed a cross-sectional design, which precludes any conclusions regarding causality or the direction of effects. Although the proposed sequential model is theoretically grounded, the assumed temporal order cannot be empirically verified with the current data. Reciprocal relationships may exist. Therefore, future research should adopt longitudinal or experimental designs to rigorously examine the causal pathways suggested by this model and to establish the temporal precedence of the variables. In addition, although Harman's single-factor test did not indicate severe common method bias, this method has inherent limitations. Future studies could employ more robust statistical techniques to control for common method variance, such as the unmeasured latent method factor approach. Second, while this study focused on the core cognitive-affective pathway, social media addiction and social anxiety may also be influenced by other factors, including personality traits and social support. Future research could incorporate additional variables into the model to enhance its explanatory power.

## 6 Conclusion and educational suggestions

By validating a serial multiple mediation model, this study delineates a network of associations linking social media addiction to social anxiety, with alienation, self-focused attention, and fear of negative evaluation acting as sequential mediators. These findings provide a detailed correlational picture of how these factors are interrelated in the digital era, suggesting a potential psychological pathway connecting behavioral dependence and emotional distress. The results enhance understanding of the etiology of social anxiety in the digital era, highlight the identifiable psychological progression from behavioral dependence to emotional disorder, and provide empirical evidence for targeted mental health interventions.

At the practical level, the findings of this study provide specific targets for prevention and intervention strategies addressing social anxiety issues associated with social media addiction. First, since alienation constitutes the initial component in the serial mediation chain, interventions should focus on assisting individuals in establishing and maintaining healthy offline interpersonal relationships while fostering a sense of community belonging to mitigate potential real-world alienation resulting from excessive media use. Second, cognitive training should emphasize modifying individuals' attentional patterns, facilitating a shift from excessive self-monitoring to external tasks and social interactions, thereby reducing self-focused attention. Finally, grounded in cognitive-behavioral therapy principles, directly addressing and restructuring maladaptive beliefs concerning negative evaluation represents a crucial approach to diminishing evaluation apprehension. Intervention programs targeting this comprehensive “alienation → self-focused attention → fear of negative evaluation” pathway may demonstrate greater efficacy than approaches limited to restricting media usage time.

## Data Availability

The raw data supporting the conclusions of this article will be made available by the authors, without undue reservation.
